# Supplementary material for: Leveraging large-scale biobank EHRs to enhance pharmacogenetics of cardiometabolic disease medications
Source: Nat Commun. 2025 Mar 25;16:2913. doi: 10.1038/s41467-025-58152-3 (PMC11937416; doi:10.1038/s41467-025-58152-3)
Supplement: Supplementary file 1 — Supplementary Information [file 41467_2025_58152_MOESM1_ESM.pdf]

Supplementary Information for

Leveraging large-scale biobank EHRs to enhance pharmacogenetics of cardiometabolic disease medications

Contents

1 **Supplementary Notes** 2

2 **Supplementary Figures** 5

List of Figures

1 Flow diagram of quality control steps. 6
2 Add-on therapy definition. 6
3 Number of individuals in each UK Biobank drug response cohort and reasons for removal. 7
4 Baseline-to-prescription start time distribution. 9
5 Post-treatment-to-prescription start time distribution. 10
6 HbA1c response to metformin, SBP response to beta blocker and HR response to beta blocker GWAS results. 11
7 SBP response to first-line antihypertensives GWAS results. 12
8 LDL-C response to statins GWAS results in the different filtering scenarios. 13
9 Total cholesterol (TC) response to statins GWAS results in the different filtering scenarios. 14
10 Number of individuals in each All of Us drug response cohort and reasons for removal. 15
11 Power analysis across ancestral groups. 16
12 Directed acyclic graph to model the genetics of drug response based on biomarker levels before and after drug treatment. 17
13 Longitudinal biomarker change GWAS in medication-naive individuals for LDL, TC and HDL. 18
14 Longitudinal biomarker change GWAS in medication-naive individuals for HbA1c, HR and SBP. 19
15 GWAS results for baseline adjusted drug response phenotypes (statin-LDL, statin-TC, statin-HDL, metformin-HbA1c, beta blocker-HR, beta blocker-SBP). 20
16 GWAS results for baseline adjusted drug response phenotypes (SBP response to first-line antihypertensives). 21
17 Baseline adjusted longitudinal biomarker change GWAS in medication-naive individuals 22

1 Supplementary Notes

Supplementary Note 1.

According to Fig. 3a, biomarker levels Y at time t can be modelled as follows:

$$Y_t = \beta_0 \cdot G_0 + \beta_E \cdot E_t + \gamma_E \cdot G_E \cdot E_t + \beta_D \cdot D_t + \gamma_D \cdot G_D \cdot D_t + \epsilon_t \tag{1}$$

where β_0 is the baseline genetic effect, G the genetics, β_E the environmental effect, E the environment, γ_E the gene-environment interaction effect, D the indicator of drug use, β_D the drug effect and γ_D the pharmacogenetic effect.

The drug response phenotype which is the difference between post-treatment Y_{t_1} and baseline Y_{t_0} biomarker levels can thus be modelled as follows:

$$Y_{t_1} - Y_{t_0} = \beta_E(E_{t_1} - E_{t_0}) + \gamma_E \cdot G_E(E_{t_1} - E_{t_0}) + \beta_D(D_{t_1} - D_{t_0}) + \gamma_D \cdot G_D(D_{t_1} - D_{t_0}) + \Delta\epsilon_{01} \tag{2}$$

$$= \beta_D + \gamma_D \cdot G_D + \delta_{01} \tag{3}$$

where baseline genetics of Y_{t_1} and Y_{t_0} cancel each other out, D_{t_1} and D_{t_0} correspond by definition to 1 and 0, respectively, and $(\beta_E + \gamma_E \cdot G_E)(E_{t_1} - E_{t_0}) + \Delta\epsilon_{01}$ are regrouped under δ_{01} assuming that interactions between genetics and changing environments are negligible (see control GWAS on longitudinal change in Supplementary Figs. 13-14). The same derivation applies to the logarithmic difference $\log(Y_{t_1}) - \log(Y_{t_0}) = \log(Y_{t_1}/Y_{t_0})$ which can be interpreted as a relative change in biomarker levels.

Adjusting drug response phenotypes or change scores for baseline biomarker levels Y_{t_0} wrongly introduces baseline genetic effects into expression 3 which results in the estimation of β_0 in addition to γ_D which we elaborate in the following.

To simplify the calculations, let us assume that all these variables (Y, G, E) are scaled to have zero mean and unit variance. When we regress Y_{t_1} onto Y_{t_0} the regression estimate $\widehat{\alpha}$ will be

$$\begin{aligned} E[\widehat{\alpha}] &= E[Y_{t_1} \cdot Y_{t_0}] = E[(\beta_0 \cdot G_0 + \beta_E \cdot E_{t_1} + \gamma_E \cdot G_E \cdot E_{t_1} + \beta_D \cdot D_{t_1} + \gamma_D \cdot G_D \cdot D_{t_1} + \epsilon_{t_1}) \\ &\quad \times (\beta_0 \cdot G_0 + \beta_E \cdot E_{t_0} + \gamma_E \cdot G_E \cdot E_{t_0} + \beta_D \cdot D_{t_0} + \gamma_D \cdot G_D \cdot D_{t_0} + \epsilon_{t_0})] \\ &= \beta_0^2 + \beta_E^2 \cdot \text{corr}(E_{t_1}, E_{t_0}) + \gamma_E^2 \cdot \text{var}(G_E) \cdot \text{corr}(E_{t_1}, E_{t_0}) \\ &= \beta_0^2 + (\beta_E^2 + \gamma_E^2) \cdot \text{corr}(E_{t_1}, E_{t_0}) \end{aligned}$$

Thus the residual from such a regression will be

$$R_{0,1} = Y_{t_1} - \widehat{\alpha} \cdot Y_{t_0} \tag{4}$$

Note that $\widehat{\alpha}$ only changes by a constant 1 if we regress the biomarker difference $Y_{t_1} - Y_{t_0}$ onto Y_{t_0} instead of Y_{t_1} onto Y_{t_0}, making these two approaches equivalent. This can be shown as follows:

$$Y_{t_1} - Y_{t_0} = \alpha' \cdot Y_{t_0} + \epsilon \tag{5}$$

Rearranging Equation 5 results in:

$$Y_{t_1} = (\alpha' + 1) \cdot Y_{t_0} + \epsilon = \alpha \cdot Y_{t_0} + \epsilon \tag{6}$$

When running a GWAS on this residual response phenotype its correlation with G_0 will be

$$\text{corr}(R_{0,1}, G_0) = E[(Y_{t_1} - \widehat{\alpha} \cdot Y_{t_0}) \cdot G_0] = \beta_0 \cdot (1 - \widehat{\alpha}) = \beta_0 \cdot (1 - \beta_0^2 - (\beta_E^2 + \gamma_E^2) \cdot \text{corr}(E_{t_1}, E_{t_0})) \tag{7}$$

Since in realistic settings $\beta_0^2 << \beta_E^2 + \gamma_E^2$ and the autocorrelation of the environment is substantial, the cubic term in β_0 is negligible and it simplifies to

$$\text{corr}(R_{0,1}, G_0) \approx \beta_0 \cdot (1 - (\beta_E^2 + \gamma_E^2) \cdot \text{corr}(E_{t_1}, E_{t_0})) \tag{8}$$

This means that when regressing the post-treatment effect on the pre-treatment effect and running a GWAS on the residuals, we expect to see a strong (spurious) genetic correlation with the genetic basis of the (time-invariant) baseline effect. Note that this correlation between the residual and the baseline genetics is identical whether we used drug-naive or pre- vs post-treated individuals. This observation further confirms that genetic "discoveries" based on residual response definitions are likely to be non-specific to the treatment.

If we examine the correlation between these residuals and the underlying drug response genetics we have

$$\text{corr}(R_{0,1}, G_D) = E[(Y_{t_1} - \widehat{\alpha} \cdot Y_{t_0}) \cdot G_0] = \gamma_D \cdot D_{t_1} - \widehat{\alpha} \cdot \gamma_D \cdot D_{t_0} \tag{9}$$

Thus, this correlation in drug-naive samples (where $D_{t_0} = D_{t_1} = 0$) is zero, but in post- vs pre-treated samples (where $D_{t_0} = 0$ and $D_{t_1} = 1$) is γ_D.

It is clear that if we simply use the post-treatment vs baseline difference, i.e. $Y_{t_1} - Y_{t_0}$, its correlation with G_0 is zero and its correlation with G_D is γ_D. Therefore, it is strongly recommended to use the simple post-treatment - baseline biomarker difference to elucidate the pure treatment-specific genetic effects.

Supplementary Note 2.

HbA1c values were either DCCT (Diabetes Control and Complications Trial) aligned (codes: 42W4. and XaERp; percentage unit) or IFCC (International Federation of Clinical Chemistry and Laboratory Medicine) aligned (42W5. and XaPbt; mmol/mol unit). For consistency, we used mmol/mol units and converted DCCT units using the NGSP/IFCC equation recommended by the National Glycohemoglobin Standardization Program (NGSP) network (`https://ngsp.org/ifcc.asp`): NGSP = [0.09148 · IFCC] + 2.152.

Supplementary Note 3.

Medication codes can correspond to multiple active ingredients taken in combination, among which the primary medication of interest. Since we cannot disentangle the effect of the primary medication compared to a second ingredient taken in combination, we filter out individuals with prescriptions corresponding to combination therapies during the study period. For statins, we eliminate combination therapies with ezetimibe and fenofibrate, for metformin, combination therapies with sitagliptin, linagliptin, saxagliptin, alogliptin, dapagliflozin, canagliflozin, empagliflozin, rosiglitazone, pioglitazone, vildagliptin and for beta blockers, combination therapies with diuretics and aspirin.

Note that this step is specific to drugs with a combined formulation and is different from the QC step where individuals taking a drug from the same medication class, but with a separate prescription code, are filtered out.

2 Supplementary Figures

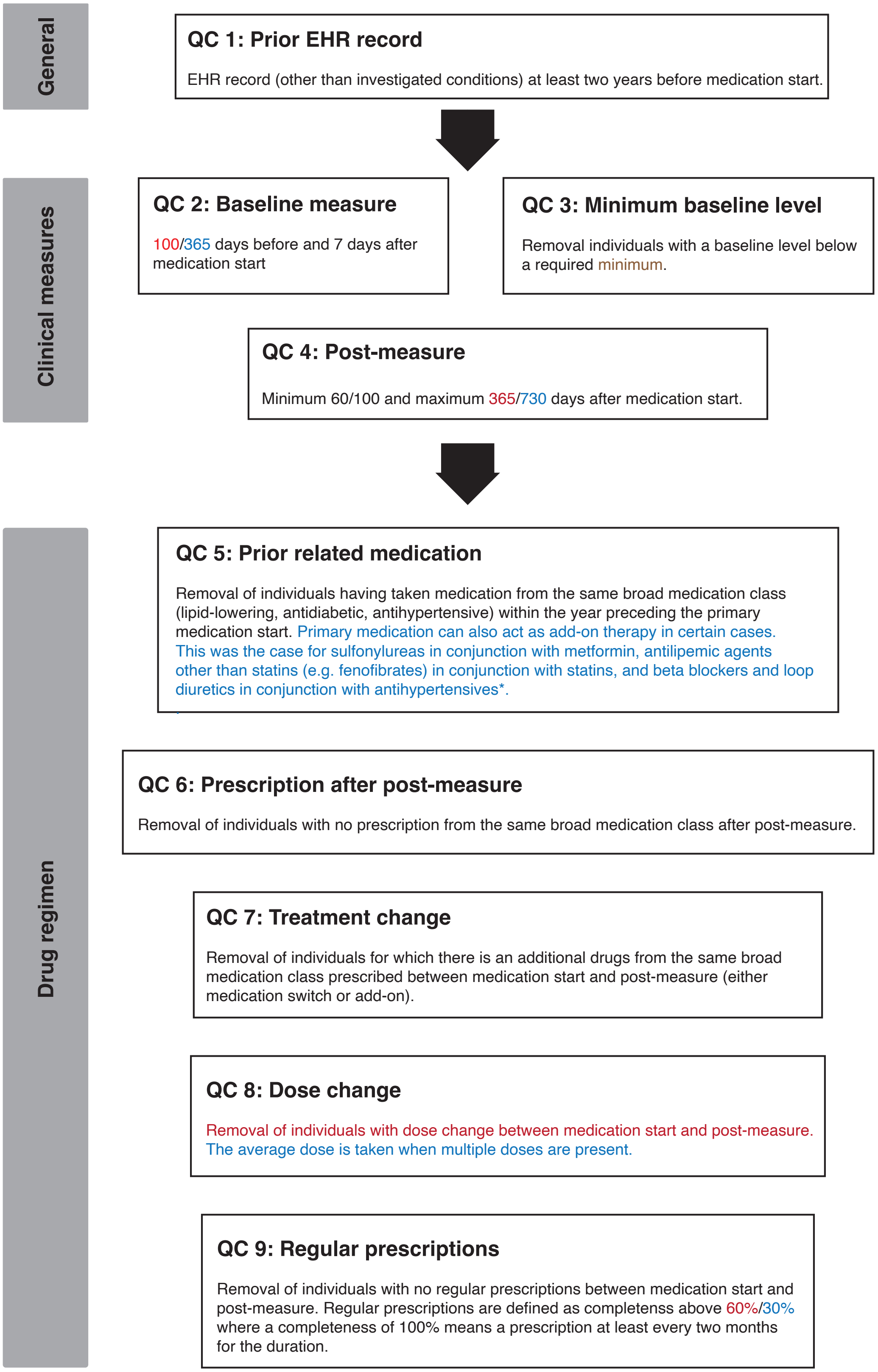

Supplementary Fig. 1: Flow diagram of quality control (QC) steps taken in the analysis of electronic health records (EHRs). After selecting individuals taking the primary medication of interest, individuals with missing biomarker measures, medication therapy changes before post-treatment measures, irregular prescriptions, or not enrolled in the health care system before the medication start were removed. Stringent filtering criteria are written in red and lenient ones in blue. Medication/phenotype-specific criteria are written in brown.

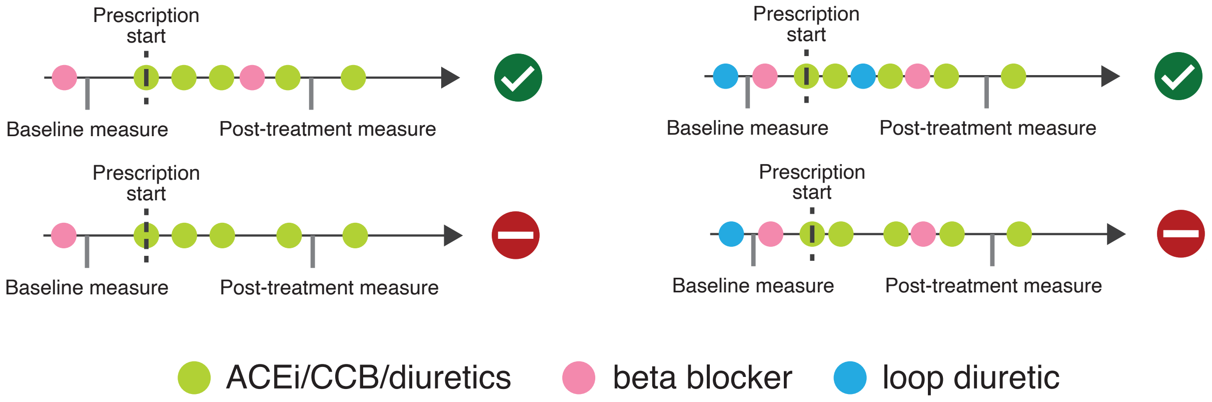

Supplementary Fig. 2: Add-on therapy definition. For antihypertensives, primary medication (ACE-inhibitor (ACEi), calcium channel blocker (CCB) and thiazide diuretics) could also act as add-on therapy to beta blockers and loop diuretics. However, medication prescribed before the primary medication start was also required to be prescribed afterwards (at least until the post-treatment measure). If the start of a beta blocker or loop diuretics medication was after the prescription start of the primary medication, this would count as "treatment change" and the individual would be removed.

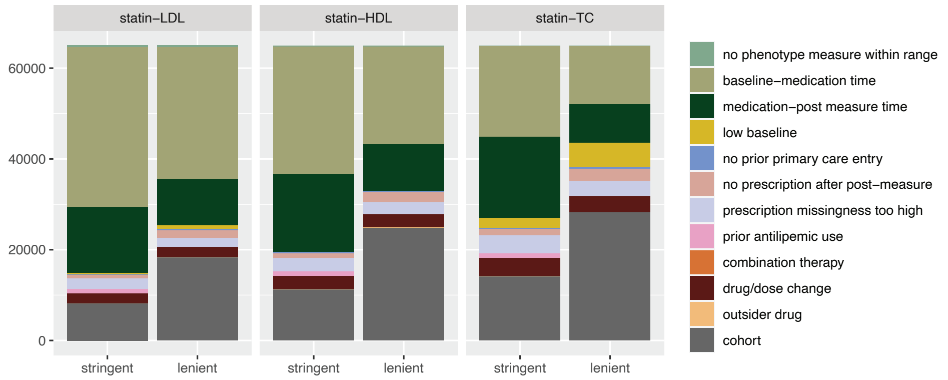
statin–LDL
statin–HDL
statin–TC
60000
40000
20000
0
stringent
lenient
no phenotype measure within range
baseline–medication time
medication–post measure time
low baseline
no prior primary care entry
no prescription after post–measure
prescription missingness too high
prior antilipemic use
combination therapy
drug/dose change
outsider drug
cohort

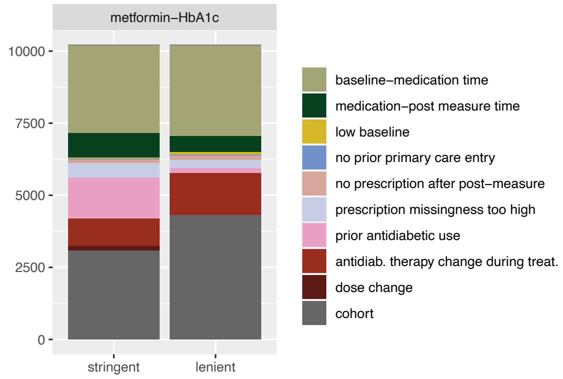
metformin–HbA1c
10000
7500
5000
2500
0
stringent
lenient
baseline–medication time
medication–post measure time
low baseline
no prior primary care entry
no prescription after post–measure
prescription missingness too high
prior antidiabetic use
antidiab. therapy change during treat.
dose change
cohort

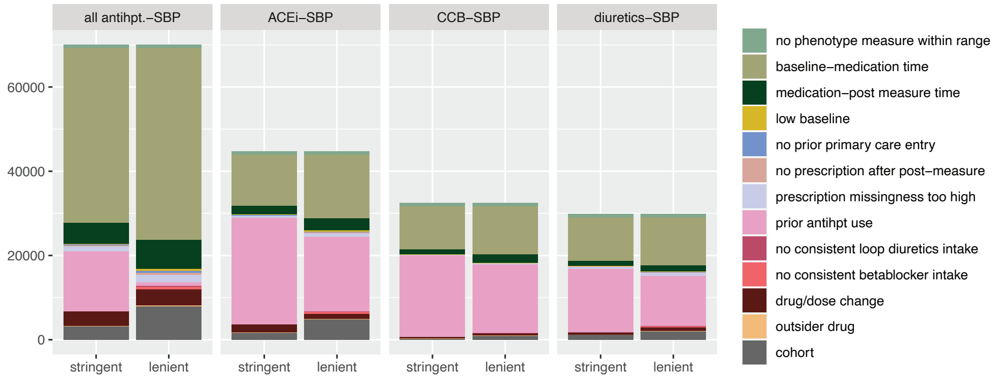
all antihpt.–SBP
ACEi–SBP
CCB–SBP
diuretics–SBP
60000
40000
20000
0
stringent
lenient
no phenotype measure within range
baseline–medication time
medication–post measure time
low baseline
no prior primary care entry
no prescription after post–measure
prescription missingness too high
prior antihpt use
no consistent loop diuretics intake
no consistent betablocker intake
drug/dose change
outsider drug
cohort

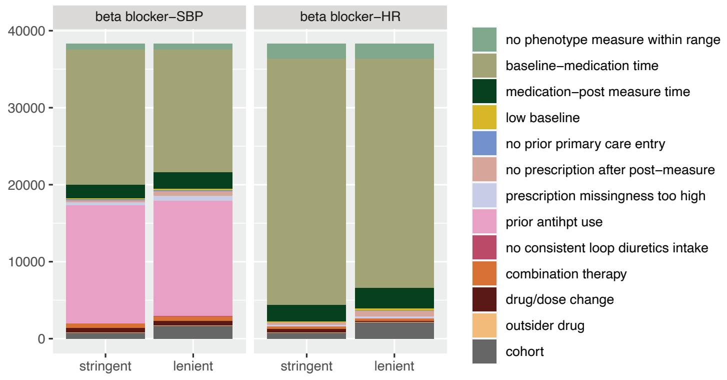
beta blocker–SBP
beta blocker–HR
40000
30000
20000
10000
0
stringent
lenient
no phenotype measure within range
baseline–medication time
medication–post measure time
low baseline
no prior primary care entry
no prescription after post–measure
prescription missingness too high
prior antihpt use
no consistent loop diuretics intake
combination therapy
drug/dose change
outsider drug
cohort

Supplementary Fig. 3: Number of individuals in each UK Biobank drug response cohort and reasons for removal (stacked barplot). The height of the bar represents the number of individuals having at least one prescription of the investigated drug. The bottom grey bar represents the number of individuals after QC steps. Note that some filtering reasons are not mutually exclusive. For instance, baseline-medication time filtering was done after checking for prior related medication. Therefore, for metformin-HbA1c in the lenient scenario, it seems that more individuals were filtered out because of baseline-medication time than in the stringent scenario. However, given that individuals with previous sulfonylureas use were excluded in the stringent, but included in the lenient filtering setting, there is a larger pool of individuals in the lenient scenario for whom baseline measures are potentially missing. The same reasoning holds for antihypertensives where individuals with prior beta blocker and loop diuretics prescriptions were included in the lenient filtering scenario (Supplementary Fig. 2). Total cholesterol (TC), antihypertensives (antihpt.), systolic blood pressure (SBP), ACE inhibitor (ACEi), calcium channel blocker (CCB), heart rate (HR).

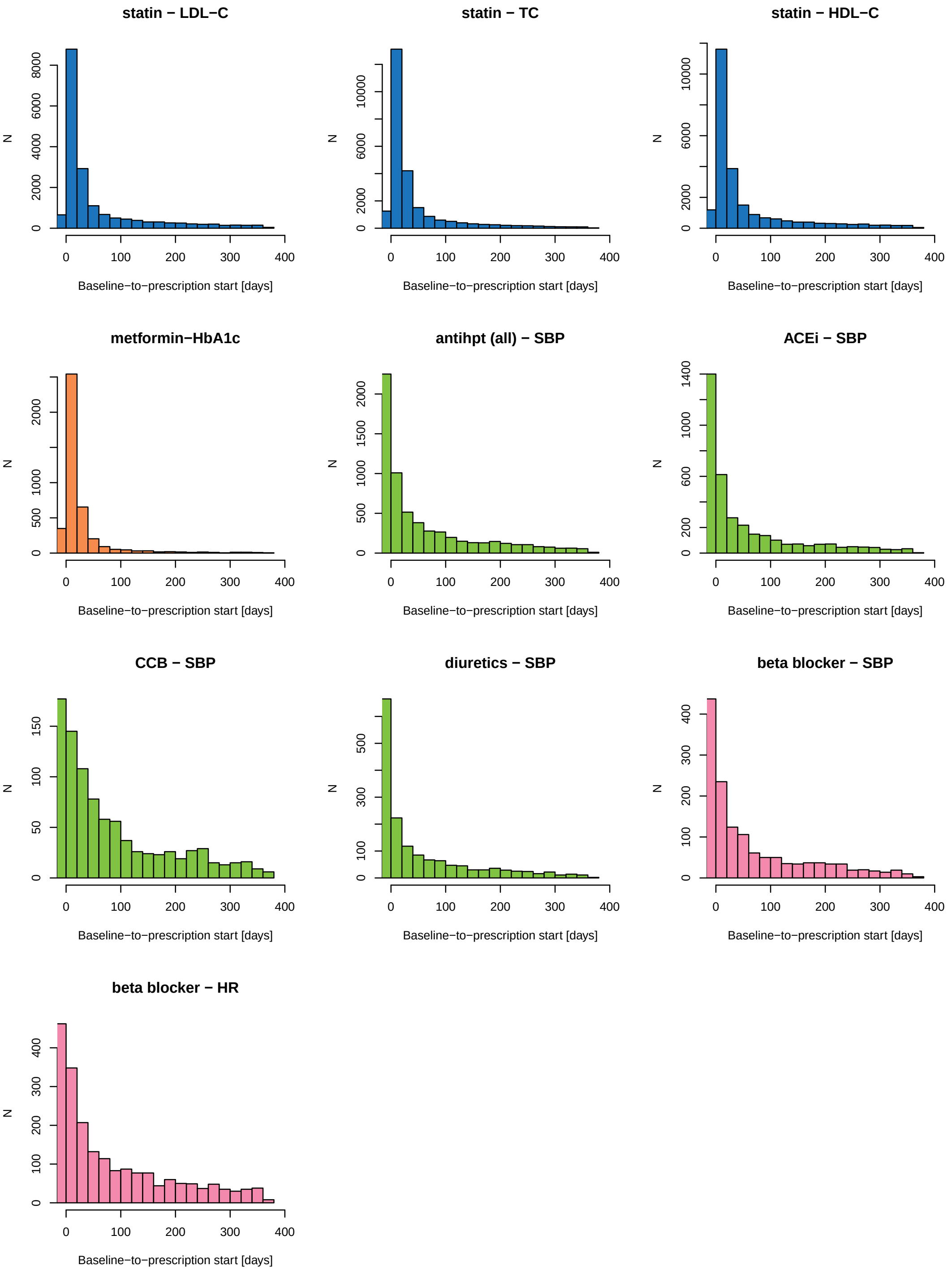

Supplementary Fig. 4: Baseline-to-prescription start time distribution. Distribution of the time between baseline measure closest to prescription start and prescription start for the ten cardiometabolic medication-biomarker pairs. In this analysis, baseline measures were restricted to occur one year before and up to seven days after prescription start (-7 on the graph). Colours represent the medication type: statin (blue), metformin (orange), first-line antihypertensives (green) and beta blocker (purple). LDL-cholesterol (LDL-C), total cholesterol (TC), HDL-cholesterol (HDL-C), antihypertensives (antihpt), systolic blood pressure (SBP), ACE inhibitors (ACEi), calcium channel blocker (CCB), heart rate (HR).

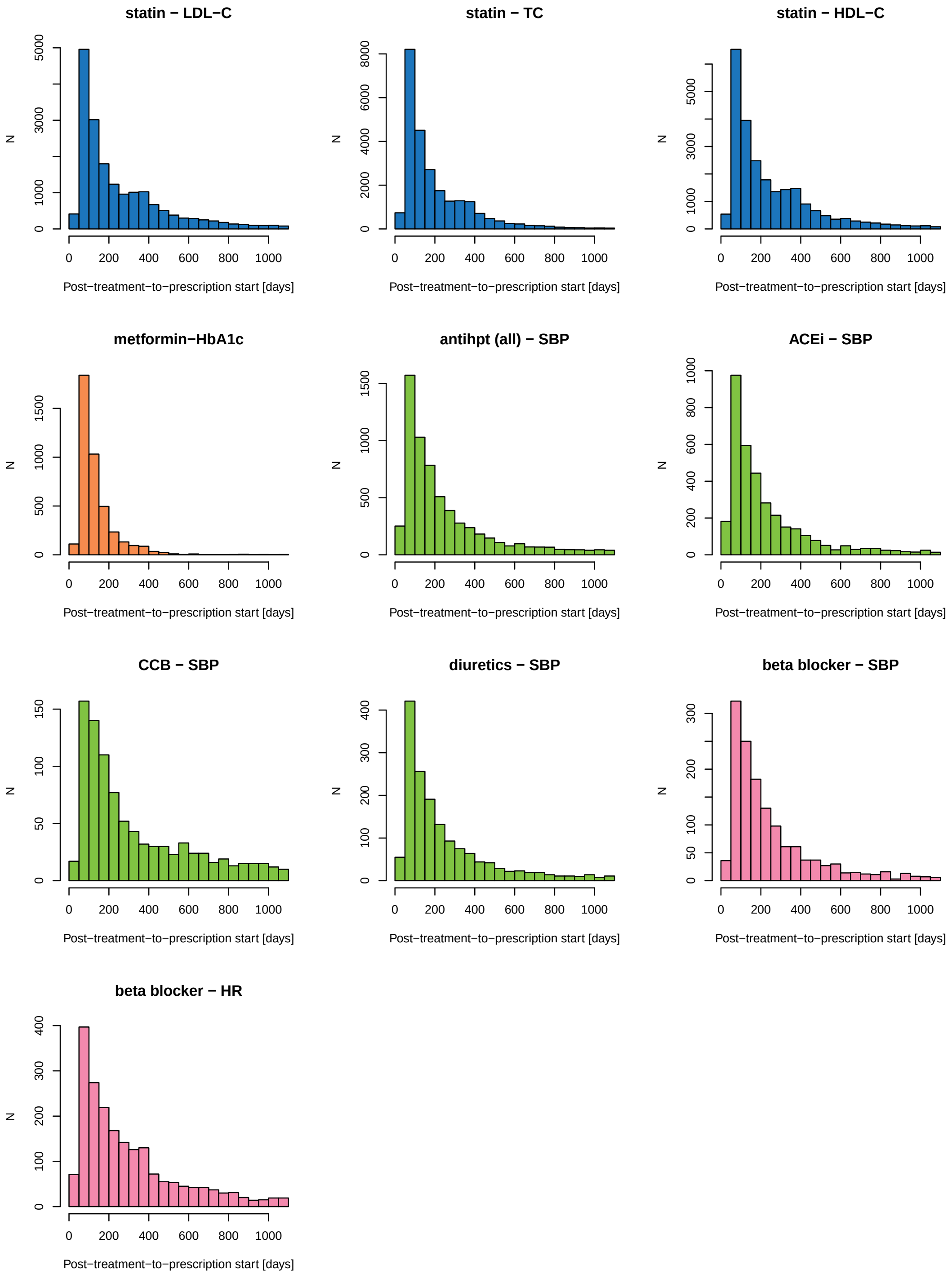

Supplementary Fig. 5: Post-treatment-to-prescription start time distribution. Distribution of the time between post-treatment measure closest to prescription start and prescription start for the ten cardiometabolic medication-biomarker pairs. In this analysis, post-treatment measures were restricted to occur 30 days and up to three years (1095 days) after prescription start. Colours represent the medication type: statin (blue), metformin (orange), first-line antihypertensives (green) and beta blocker (purple). LDL-cholesterol (LDL-C), total cholesterol (TC), HDL-cholesterol (HDL-C), antihypertensives (antihpt), systolic blood pressure (SBP), ACE inhibitors (ACEi), calcium channel blocker (CCB), heart rate (HR).

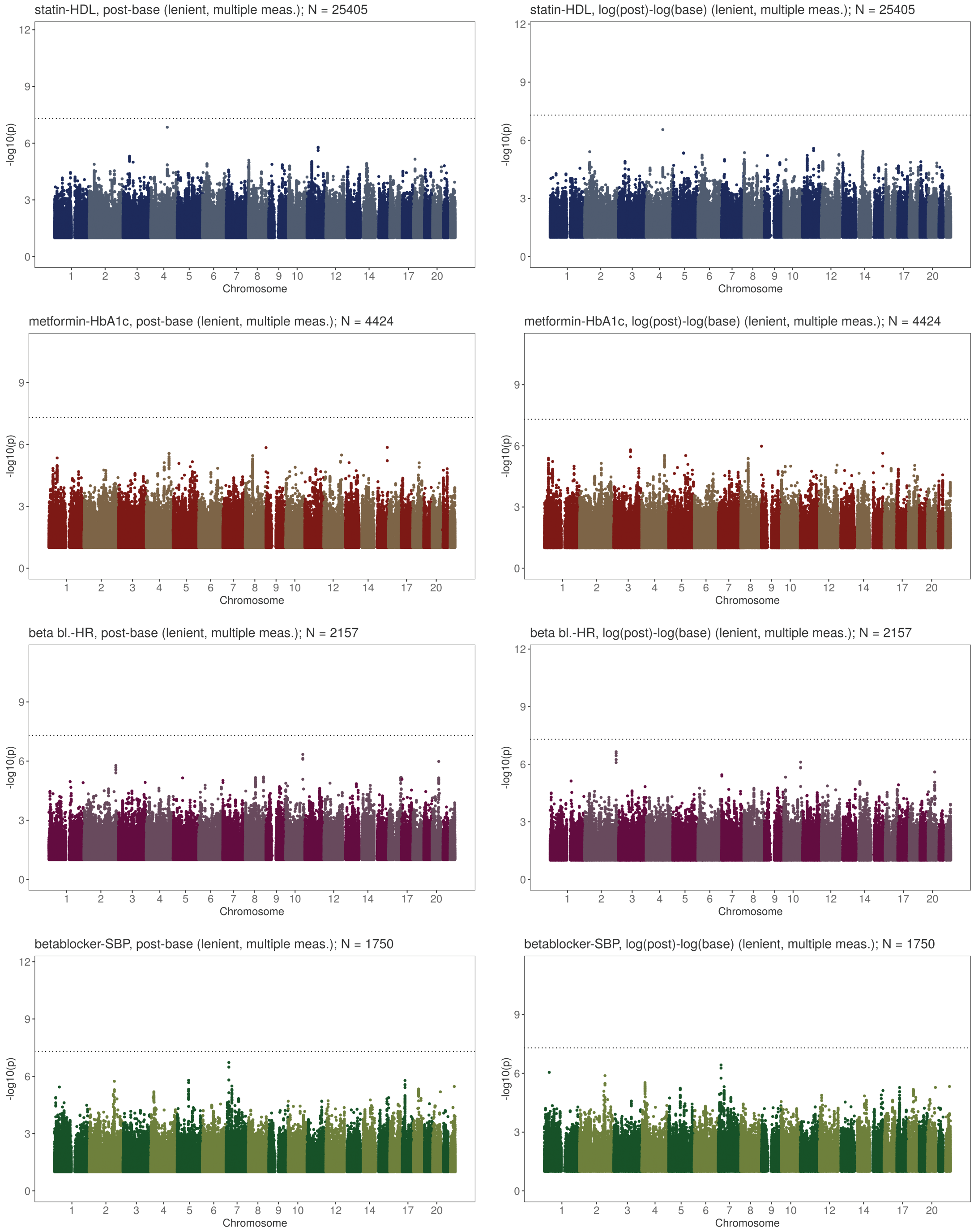

Supplementary Fig. 6: HbA1c response to metformin, SBP response to beta blocker and HR response to beta blocker GWAS results. Plots on the left show GWAS results for the absolute biomarker (post-baseline level) and plots on the right the results for the logarithmic relative (log(post)-log(base)) difference. GWAS were performed using a linear additive model, with a two-sided test of association. GWAS results correspond to the lenient filtering scenarios with average baseline and post-treatment values over multiple measures if available. Genome-wide significant loci are annotated with the closest gene. The horizontal line denotes genome-wide significance (p-value $<$ 5e-8). Colours represent the medication type: statin (blue), metformin (orange), first-line antihypertensives (green) and beta blocker (purple).

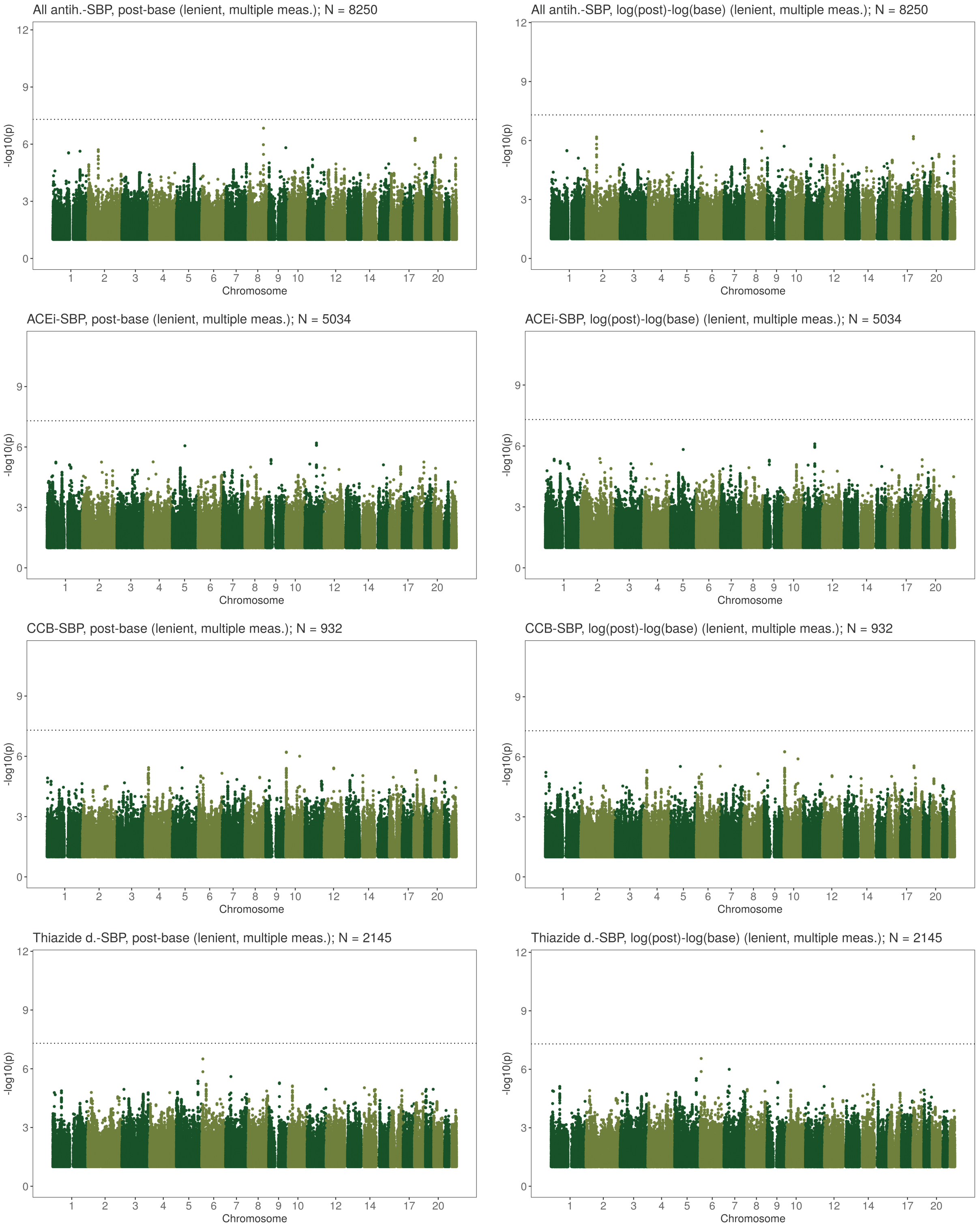

Supplementary Fig. 7: SBP response to first-line antihypertensives GWAS results. Plots on the left show GWAS results for the absolute biomarker (post-baseline level) and plots on the right the results for the logarithmic relative (log(post)-log(base)) difference. GWAS were performed using a linear additive model, with a two-sided test of association. GWAS results correspond to the lenient filtering scenarios with average baseline and post-treatment values over multiple measures if available. Genome-wide significant loci are annotated with the closest gene. The horizontal line denotes genome-wide significance (p-value $<$ 5e-8).

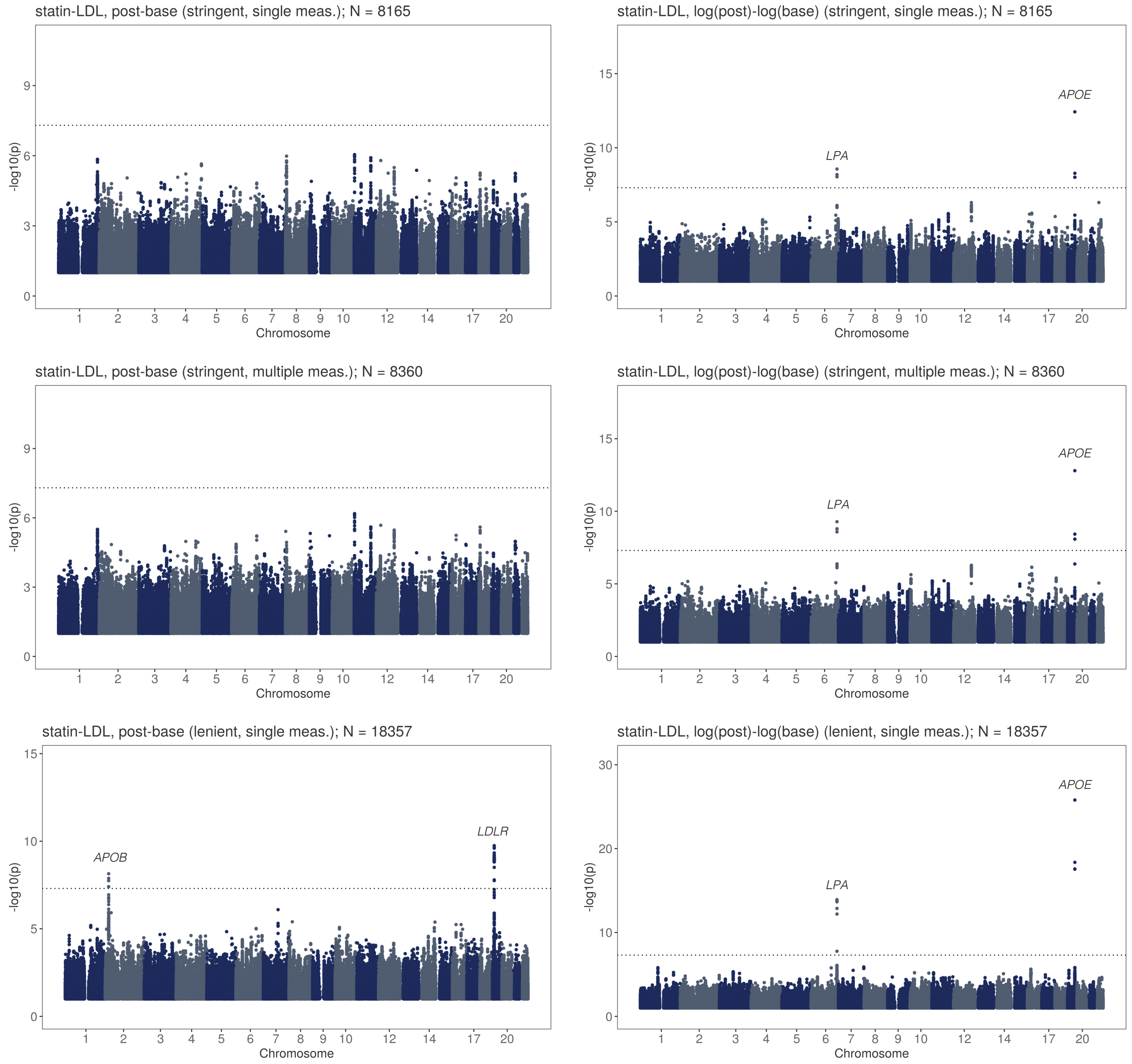

Supplementary Fig. 8: LDL-C response to statins GWAS results in the different filtering scenarios. Plots on the left show GWAS results for the absolute biomarker (post-baseline level) and plots on the right the results for the logarithmic relative (log(post)-log(base)) difference. GWAS were performed using a linear additive model, with a two-sided test of association. For stringent and lenient filtering scenarios, single baseline and post-treatment measures and average values over multiple measures, if available, were tested. Significant association signals are provided in Supplementary Data 8. Results for lenient filtering and multiple measures are shown in Fig. 3. Genome-wide significant loci are annotated with the closest gene. The horizontal line denotes genome-wide significance (p-value $< 5e-8$).

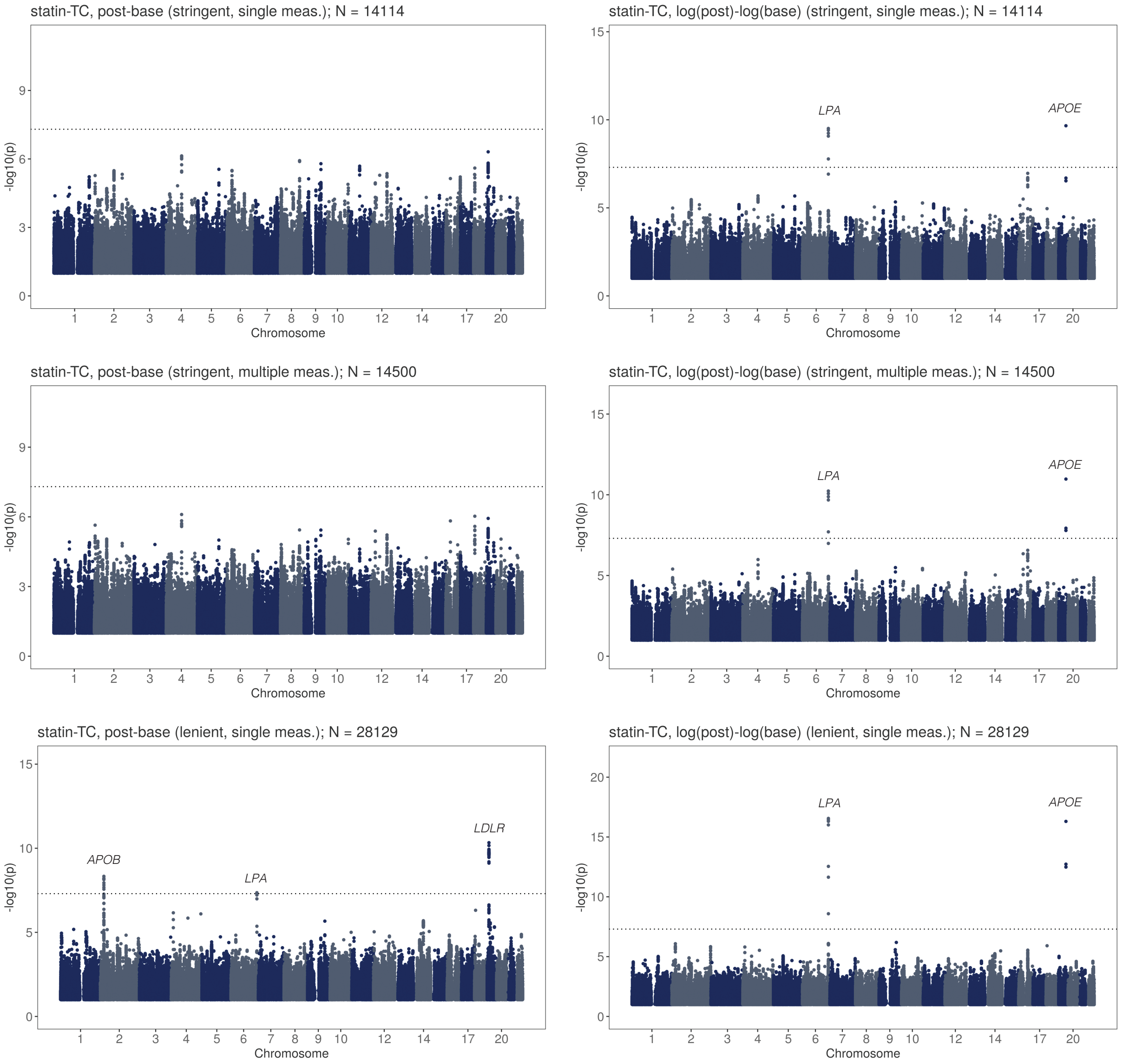

Supplementary Fig. 9: Total cholesterol (TC) response to statins GWAS results in the different filtering scenarios. Plots on the left show GWAS results for the absolute biomarker (post-baseline level) and plots on the right the results for the logarithmic relative (log(post)-log(base)) difference. GWAS were performed using a linear additive model, with a two-sided test of association. For stringent and lenient filtering scenarios, single baseline and post-treatment measures and average values over multiple measures, if available, were tested. Significant association signals are provided in Supplementary Data 8. Results for lenient filtering and multiple measures are shown in Fig. 3. Genome-wide significant loci are annotated with the closest gene. The horizontal line denotes genome-wide significance (p-value $<$ 5e-8).

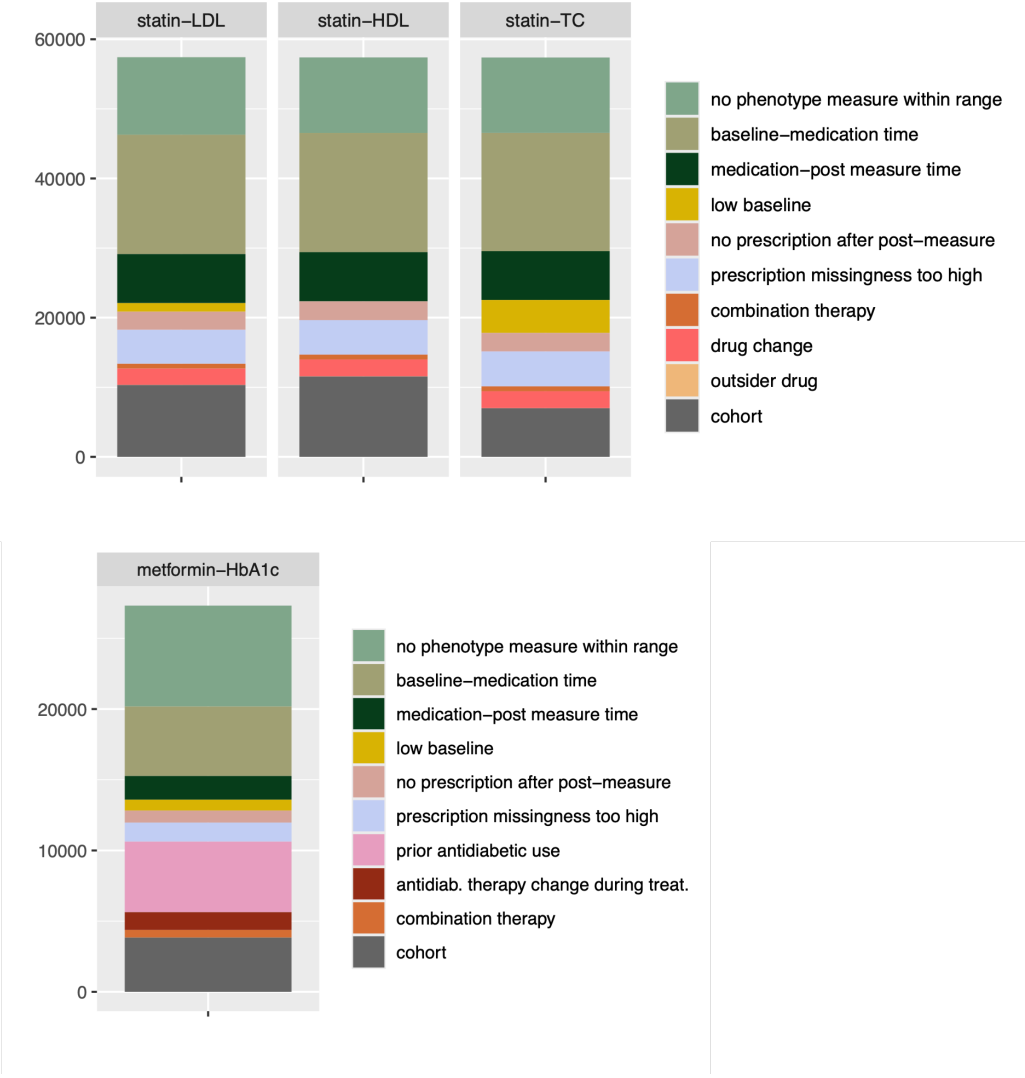

Supplementary Fig. 10: Number of individuals in each All of Us drug response cohort and reasons for removal (stacked barplot). The height of the bar represents the number of individuals having at least one prescription of the investigated drug. The bottom grey bar represents the number of individuals after QC steps. Note some filtering reasons are not mutually exclusive. Total cholesterol (TC).

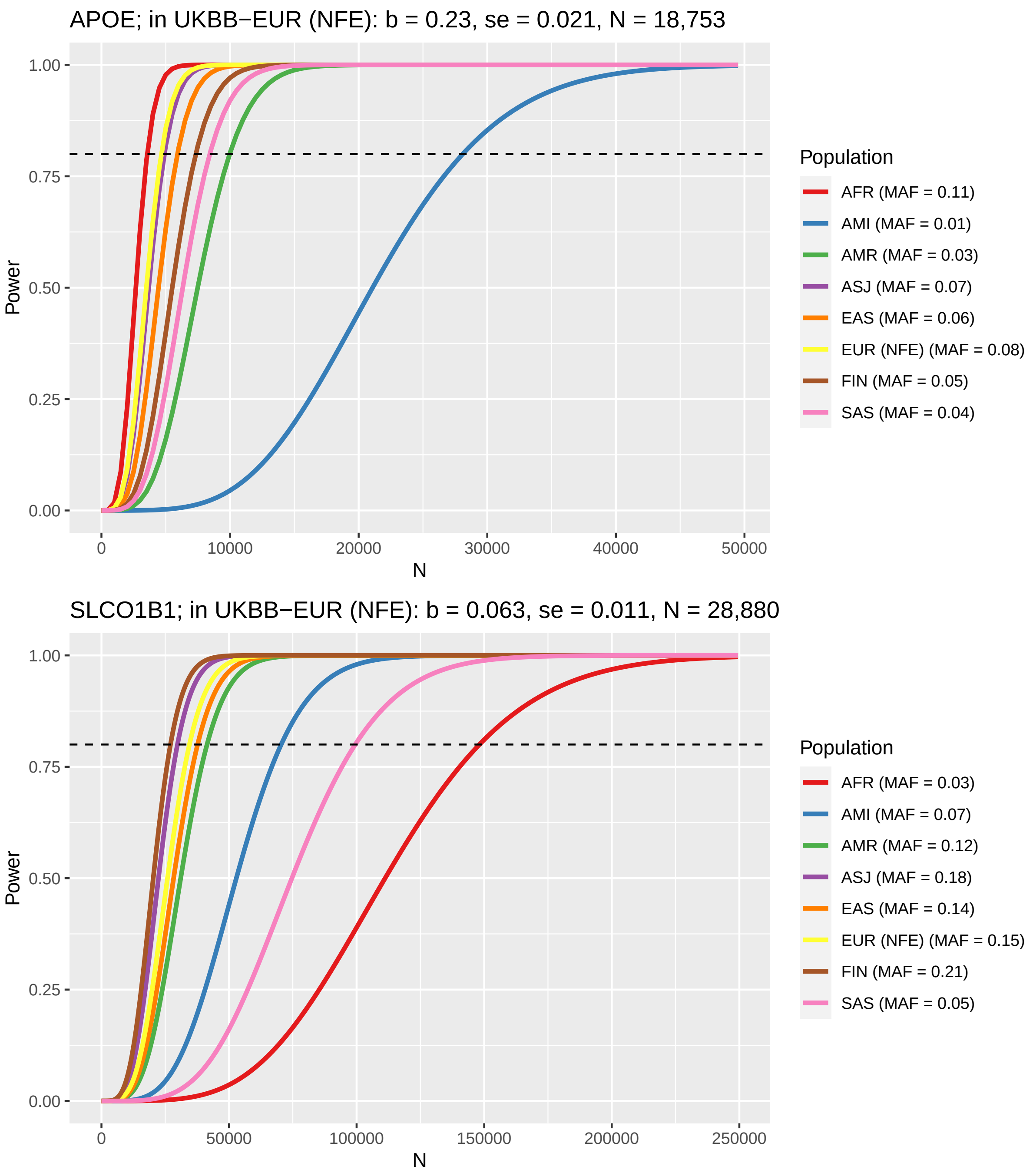

Supplementary Fig. 11: Power analysis across ancestral groups. Power analyses to determine the sample size needed to detect PGx signals at genome-wide significance (two-sided test statistic) in different ancestral groups where minor allele frequencies (MAFs) were obtained from the gnomAD v4.1.0 resource. The effect sizes for the *APOE* and *SLCO1B1* pharmacogenetic signals are based on the effect sizes obtained in the discovery analyses in participants of European ancestry (Non-Finnish; EUR (NFE)) in the UK Biobank (UKBB). The dashed horizontal line indicates 80% power.

b = effect size; se = standard error; N = sample size; AFR = African/African American; AMI = Amish; AMR = Admixed American; ASJ = Ashkenazi Jewish; EAS = East Asian; FIN = Finnish; EUR (NFE) = Non-Finnish European; SAS = South Asian

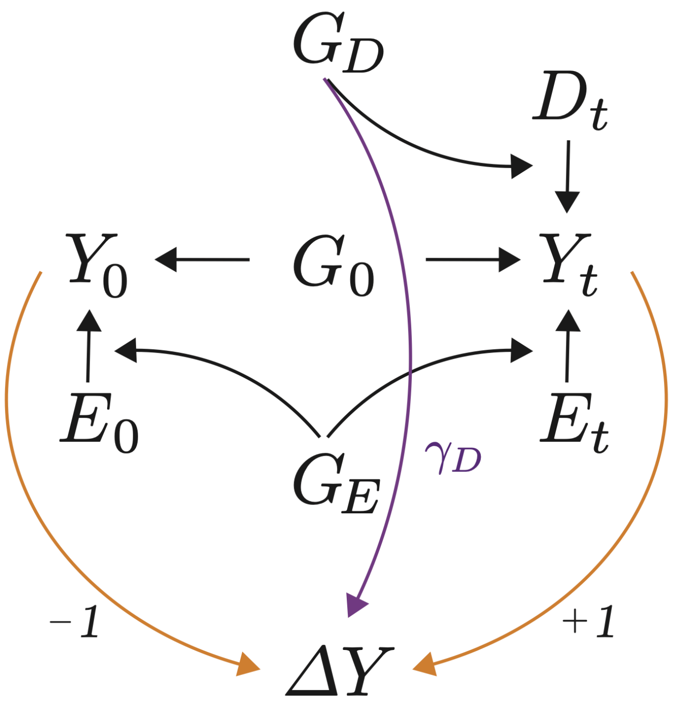

Supplementary Fig. 12: Directed acyclic graph (DAG) to model the genetics of drug response based on biomarker levels before and after drug treatment. This figure accompanies Fig. 3 in the main text. Biomarker levels Y at time t can be influenced by baseline genetics G_0, environment E and gene-environment interactions ($G_E \cdot E$), and drug status D and pharmacogenetic interactions ($G_D \cdot D$; Fig. 3). Drug response phenotypes ΔY modelled as the difference of post-treatment (t = t; +1) and baseline (t = 0; -1) levels (orange) allow the estimation of the pharmacogenetic effect γ_D (purple). The DAG illustrates how baseline genetics cancels out when taking the difference of biomarker levels, whereas the pharmacogenetic effect γ_D only acts through the post-treatment pathway.

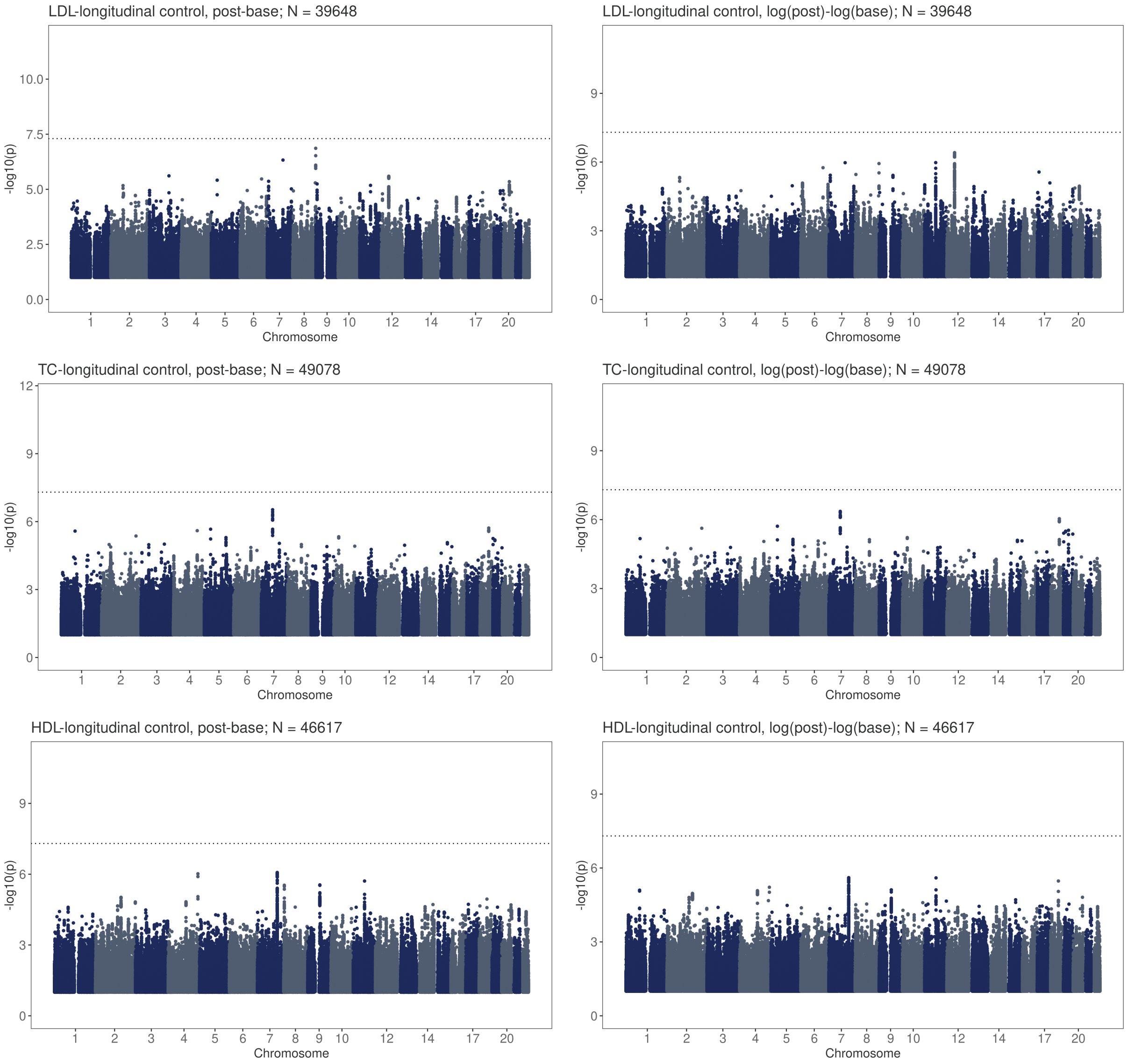

Supplementary Fig. 13: Longitudinal biomarker change GWAS in medication-naive individuals for LDL, TC and HDL. Plots on the left show GWAS results for the absolute biomarker (post-baseline level) and plots on the right the results for the logarithmic relative (log(post)-log(base)) difference. GWAS were performed using a linear additive model, with a two-sided test of association. Genome-wide significant loci are annotated with the closest gene. The horizontal line denotes genome-wide significance (p-value $< 5e-8$).

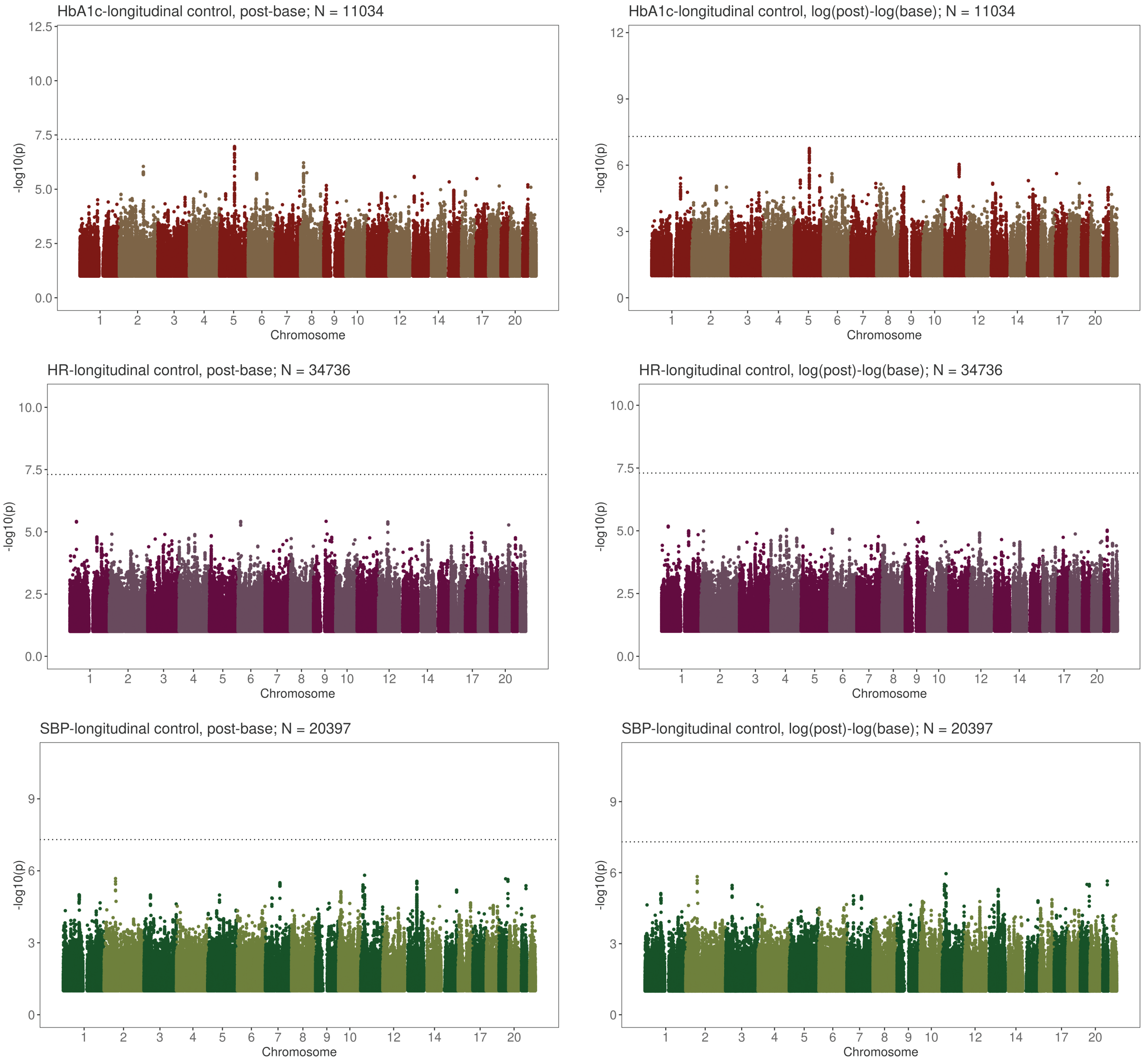

Supplementary Fig. 14: Longitudinal biomarker change GWAS in medication-naive individuals for HbA1c, HR and SBP. Plots on the left show GWAS results for the absolute biomarker (post-baseline level) and plots on the right the results for the logarithmic relative (log(post)-log(base)) difference. GWAS were performed using a linear additive model, with a two-sided test of association. Genome-wide significant loci are annotated with the closest gene. The horizontal line denotes genome-wide significance (p-value $<$ 5e-8). Colours represent the medication type associated with the assessed biomarker: HbA1c (orange), systolic blood pressure (SBP, green) and heart rate (HR, purple).

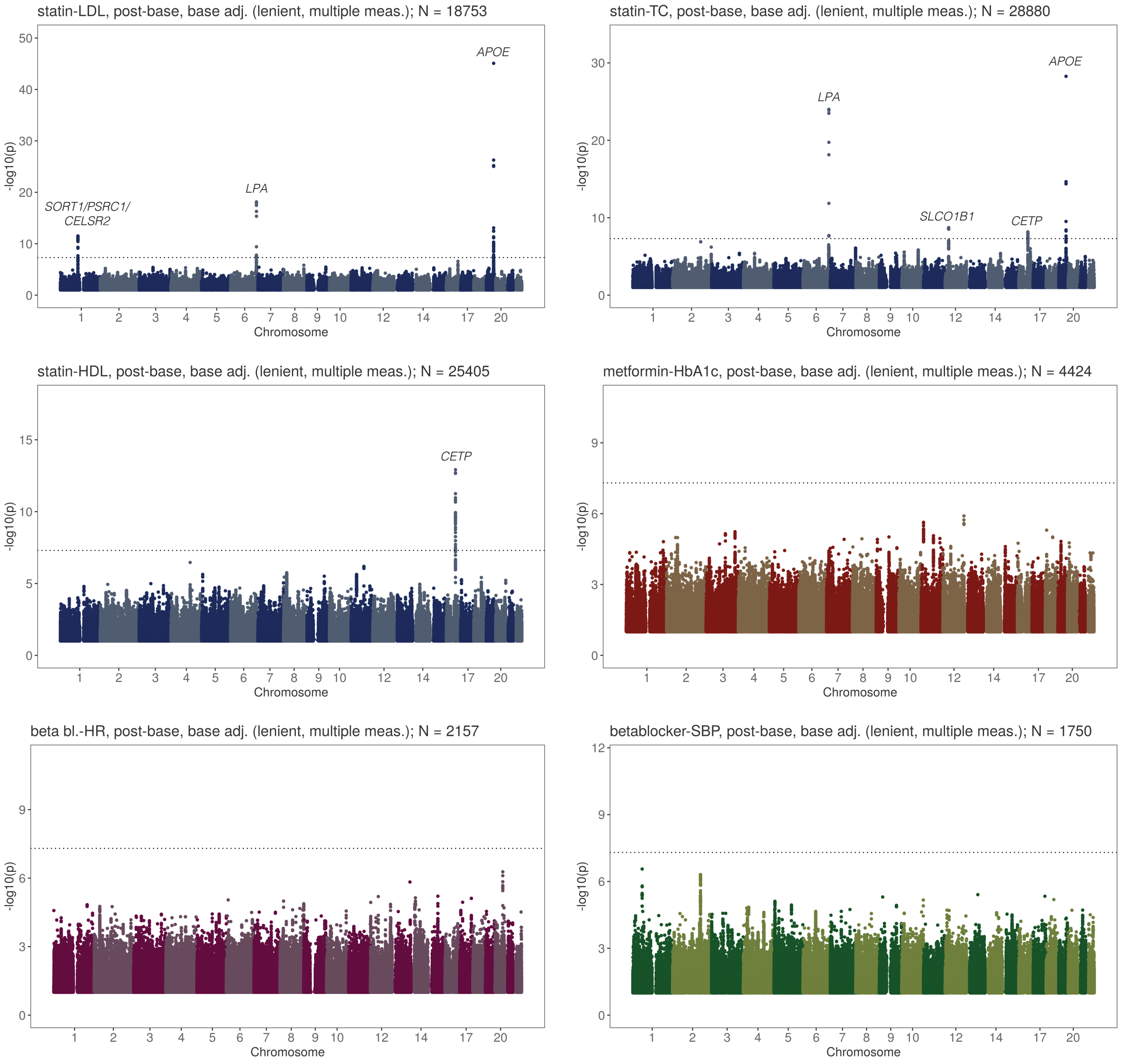

Supplementary Fig. 15: GWAS results for baseline adjusted drug response phenotypes (statin-LDL, statin-TC, statin-HDL, metformin-HbA1c, beta blocker-HR, beta blocker-SBP). GWAS were performed using a linear additive model, with a two-sided test of association. GWAS results correspond to the lenient filtering scenarios with average baseline and post-treatment values over multiple measures if available. Genome-wide significant loci are annotated with the closest gene. The horizontal line denotes genome-wide significance (p-value $<$ 5e-8). Significant association signals are provided in Supplementary Data 14. Colours represent the medication type: statin (blue), metformin (orange), first-line antihypertensives (green) and beta blocker (purple).

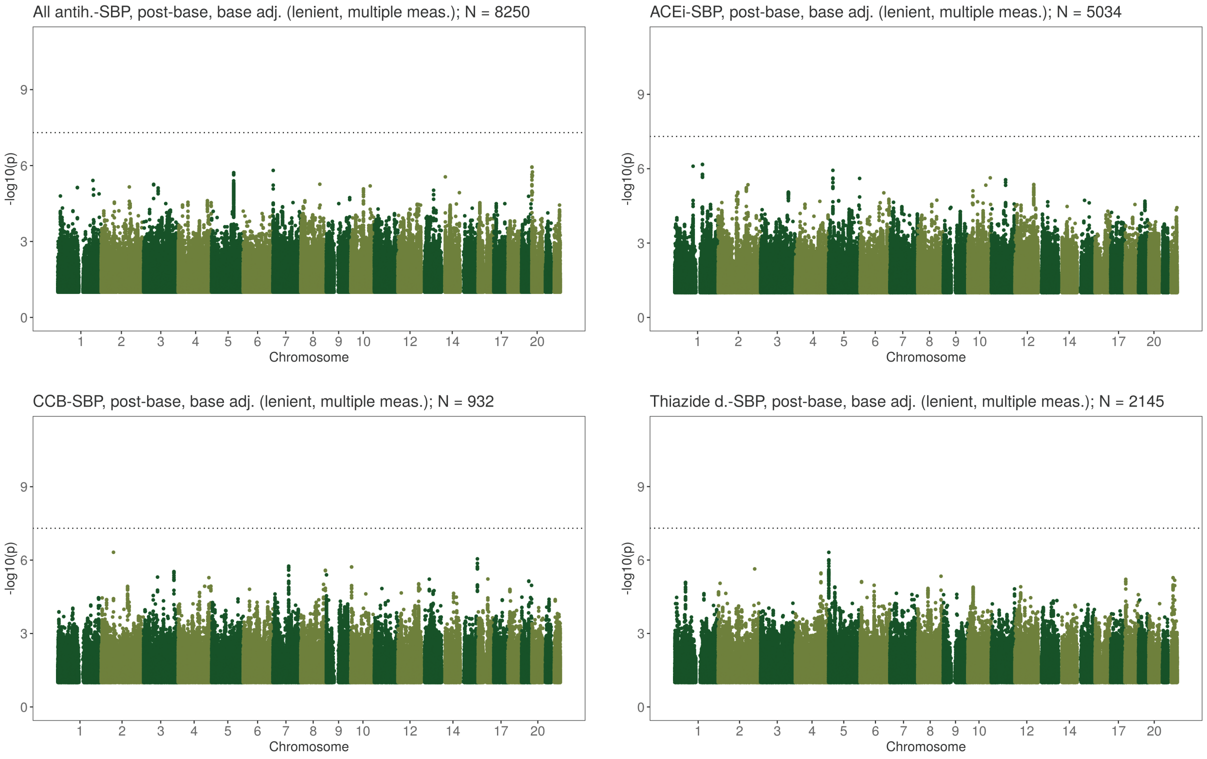

Supplementary Fig. 16: GWAS results for baseline adjusted drug response phenotypes (SBP response to first-line antihypertensives). GWAS were performed using a linear additive model, with a two-sided test of association. GWAS results correspond to the lenient filtering scenarios with average baseline and post-treatment values over multiple measures if available. Genome-wide significant loci are annotated with the closest gene. The horizontal line denotes genome-wide significance (p-value $<$ 5e-8).

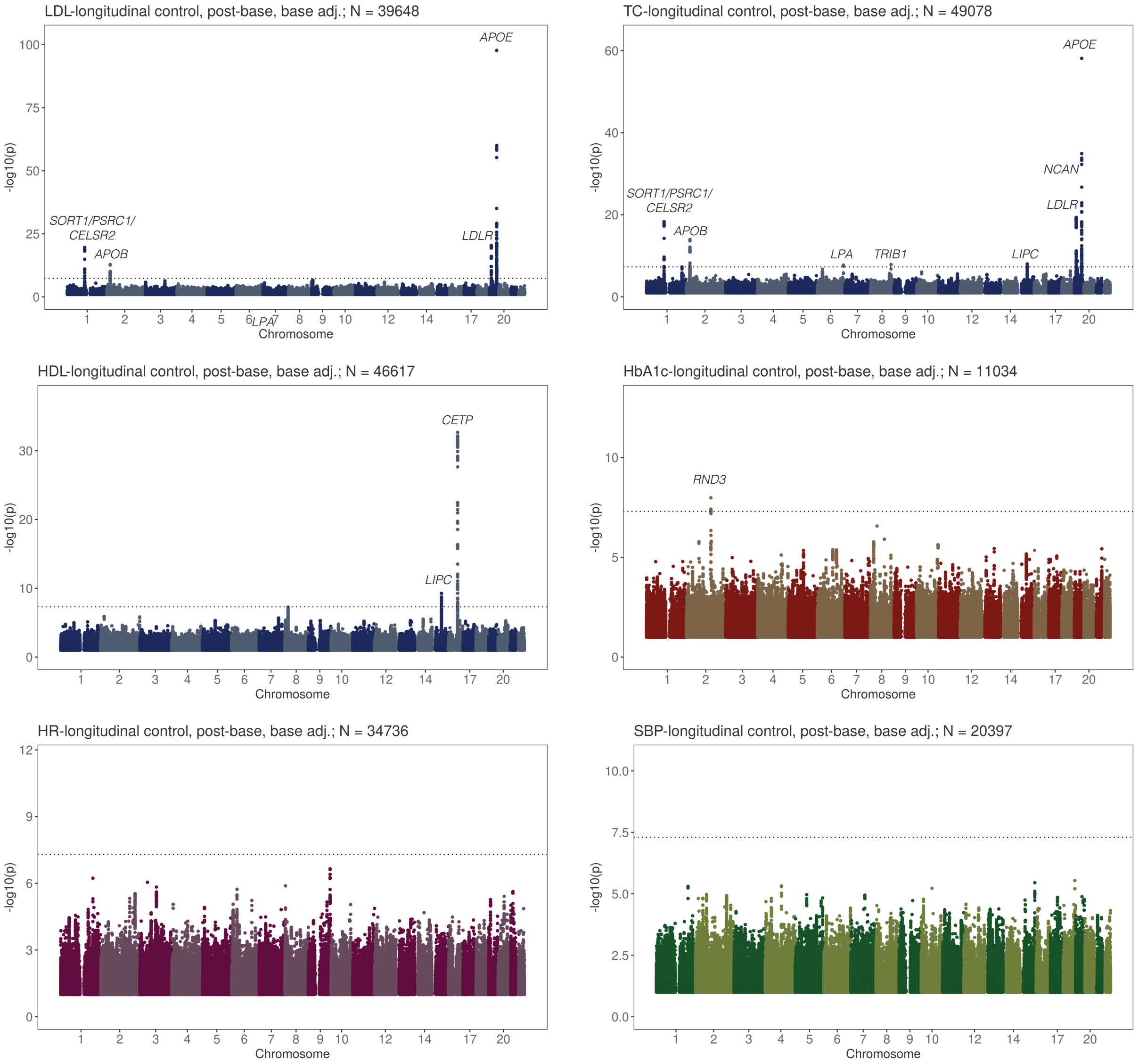

Supplementary Fig. 17: Baseline adjusted longitudinal biomarker change GWAS in medication-naive individuals. GWAS were performed using a linear additive model, with a two-sided test of association. Genome-wide significant loci are annotated with the closest gene. The horizontal line denotes genome-wide significance (p-value $<$ 5e-8). Significant association signals are provided in Supplementary Data 15. Colours represent the medication type associated with the assessed biomarker: LDL cholesterol (LDL-C, blue), total cholesterol (TC, blue), HDL-cholesterol (blue), HbA1c (orange), systolic blood pressure (SBP, green) and heart rate (HR, purple).
