# Supplementary material for: Leveraging large-scale biobank EHRs to enhance pharmacogenetics of cardiometabolic disease medications
Source: Nat Commun. 2025 Mar 25;16:2913. doi: 10.1038/s41467-025-58152-3 (PMC11937416; doi:10.1038/s41467-025-58152-3)
Supplement: Supplementary file 2 — Description of Additional Supplementary Files [file 41467_2025_58152_MOESM2_ESM.pdf]

Description of Additional Supplementary Files

Supplementary Data 1. Read V2 and CTV3 codes of clinical measures.

Supplementary Data 2. BNF and read V2 medication codes.

Supplementary Data 3. Drug response phenotype filtering criteria and cohort-specific covariates.

Supplementary Data 4. Drug response cohort study characteristics in the UK Biobank. Baseline and post-treatment levels, as well as time with respect to prescription start, correspond to the closest measures to the prescription start (single measure scenario). Minor deviations in sample size compared to the scenario with average values over multiple measures can arise as a result of the prescription completeness filter. When increasing the post-treatment period (which is the case when using multiple measures), additional prescriptions can increase the prescription completeness percentage to the required completeness threshold.

Supplementary Data 5. Drug response cohort study characteristics in the UK Biobank: starting drug type and dose statistics. Starting drug type and dose in each of the ten drug response cohorts (default scenario of lenient filtering).

Supplementary Data 6. Effect of prescription start-to-post-measure time and prescription regularity on biomarker difference. Effect sizes (beta) are on the standardized scale (SD/SD). A positive effect of the post-measure time means that a later first measure following prescriptions results in a decreased biomarker difference (i.e. smaller Delta). A negative effect of the prescription completeness (the presence of a prescription at least every two months for the duration of the post-treatment period) means that missing prescriptions result in a decreased biomarker difference (i.e. smaller Delta)**.** Associations stem from a joint model and are adjusted for sex, age and drug-specific covariates (Supplementary Data 3). P-values stem from a two-sided test of association.

Supplementary Data 7. Control cohort study characteristics.

Supplementary Data 8. Significant loci in pharmacogenetics GWAS in different filtering scenarios. GWAS were performed using a linear additive model, with a two-sided test of association.

Supplementary Data 9. Drug response cohort study characteristics in the All of Us research program.

Supplementary Data 10. Allele frequencies of identified pharmacogenetic signals across genetic ancestry groups. Identified pharmacogenetic signals (main Table 1, identified with the default lenient filtering strategy using multiple biomarker measures) and the allele

frequencies across genetic ancestry groups extracted from gnomAD v4.1.0.
Chr, chromosome; EAF, frequency of effect allele; post-base, absolute biomarker difference; log(post/base), logarithmic (relative) biomarker difference; Genetic ancestry groups: AFR = African/African American; AMI = Amish; AMR = Admixed American; ASJ = Ashkenazi Jewish; EAS = East Asian; FIN = Finnish; EUR (NFE) = Non-Finnish European; SAS = South Asian

Supplementary Data 11. Literature replication analysis of antihypertensives. Genetic predictors of drug response to antihypertensives that were reported by Oliveira-Paula et al., 2019 (PMID: 31819590) in sample sizes exceeding N > 300. Corresponding significance levels (two-sided test) were retrieved from the EHR-derived genetic analyses in the UK Biobank (UKBB).

Supplementary Data 12. Number of individuals and numerical values in each baseline, longitudinal change and drug response stratum of Figure 3. The baseline group combines statin-free controls and statin users (simvastatin 40mg corresponding to the largest starting statin type-dose group), and the mean and standard deviation (sd) within each stratum corresponds to the sex and age adjusted baseline level.
Longitudinal change phenotypes (control individuals) and drug response phenotypes (statin users) are also adjusted for sex and age as well as for baseline if indicated. The numerical values (mean, sd) within the longitudinal-control and drug response group correspond to these adjusted follow-up measures.
Prior adjustments, baseline levels and drug response/longitudinal change phenotypes were standardized to have a mean of 0 and standard deviation of 1.

Supplementary Data 13. Genotype regression coefficients in each baseline, longitudinal change and drug response group of Figure 3. Genotype regression coefficients (b) with baseline lipid levels, longitudinal change in controls and drug response phenotypes were derived through regression of the standardized outcome measures on the genotype dosage adjusted for sex and age as well as baseline levels if indicated (two-sided test of association).
For a homogeneous statin user group only those starting with simvastatin 40mg are considered which corresponds to the largest starting statin type-dose group.

Supplementary Data 14. Significant loci in pharmacogenetics GWAS adjusted for baseline. GWAS were performed using a linear additive model, with a two-sided test of association.

Supplementary Data 15. Significant loci in longitudinal control GWAS adjusted for baseline. GWAS were performed using a linear additive model, with a two-sided test of association.

Supplementary Data 16. Drug response associations with PRS. Associations with PRS are calculated for the absolute post-treatment and baseline level (post-base) and logarithmic relative (log(post)-log(base)) difference. Effect sizes (b) correspond to the effect in the respective unit for 1SD increase in PRS. As the effect sizes of the log differences on their original scale (unit-less) were very small they approximate relative change, and thus were multiplied by 100 to be interpreted as percentage changes. Standardized effect sizes (b_std) correspond to an SD change for 1SD increase in PRS. A negative signs means that increased PRS increases treatment efficacy (i.e., larger biomarker difference compared to low PRS). All

associations are adjusted for sex, age and drug-specific covariates (Supplementary Data 3). P-values stem from a two-sided association test.

Supplementary Data 17. Genetic correlations between drug response, baseline and disease progression traits. No genetic correlations can be calculated for traits with a negative heritability estimate and corresponding fields report NA. h2_1, h2_2: heritability of trait 1 and 2, respectively; se_h2_1, se_h2_2: corresponding standard errors.

Supplementary Data 18. Number of individuals and numerical values in each LDL-C baseline, LDL-C PRS and APOE stratum (Figure 4c). Post-treatment levels within each stratum (units are in mmol/L), where stratification occured on 1) baseline levels, 2) PRS and 3) APOE genotype.
